# Recommendations for the nomenclature of enteroviruses and rhinoviruses

**DOI:** 10.1007/s00705-019-04520-6

**Published:** 2020-01-25

**Authors:** P. Simmonds, A. E. Gorbalenya, H. Harvala, T. Hovi, N. J. Knowles, A. M. Lindberg, M. S. Oberste, A. C. Palmenberg, G. Reuter, T. Skern, C. Tapparel, K. C. Wolthers, P. C. Y. Woo, R. Zell

**Affiliations:** 1 Nuffield Department of Experimental Medicine, University of Oxford, Oxford, UK; 2 Department of Medical Microbiology, Leiden University Medical Center, Leiden, The Netherlands; 3 National Microbiology Service, NHS Blood and Transplant, London, UK; 4 Finnish Institute for Health and Welfare, Helsinki, Finland; 5 The Pirbright Institute, Pirbright, Woking, UK; 6 Department of Chemistry and Biomedical Sciences, Linnaeus University, Kalmar, Sweden; 7 Division of Viral Diseases, Centers for Disease Control and Prevention, Atlanta, GA USA; 8 Department of Biochemistry, Institute for Molecular Virology, Madison, WI USA; 9 Department of Medical Microbiology and Immunology, Medical School, University of Pécs, Pecs, Hungary; 10 Max Perutz Labs, Medical University of Vienna, Vienna, Austria; 11 Department of Microbiology and Molecular Medicine, Faculty of Medicine, University of Geneva, Geneva, Switzerland; 12 Department of Medical Microbiology, Academic Medical Center, University of Amsterdam, Amsterdam, The Netherlands; 13 Department of Microbiology, The University of Hong Kong, Hong Kong SAR, China; 14 Division of Experimental Virology, Institute of Medical Microbiology, Jena University Hospital, Jena, Germany

## Abstract

Enteroviruses (EVs) and rhinoviruses (RVs) are significant pathogens of humans and are the subject of intensive clinical and epidemiological research and public health measures, notably in the eradication of poliovirus and in the investigation and control of emerging pathogenic EV types worldwide. EVs and RVs are highly diverse in their antigenic properties, tissue tropism, disease associations and evolutionary relationships, but the latter often conflict with previously developed biologically defined terms, such as “coxsackieviruses”, “polioviruses” and “echoviruses”, which were used before their genetic interrelationships were understood. This has created widespread formatting problems and inconsistencies in the nomenclature for EV and RV types and species in the literature and public databases. As members of the International Committee for Taxonomy of Viruses (ICTV) *Picornaviridae* Study Group, we describe the correct use of taxon names for these viruses and have produced a series of recommendations for the nomenclature of EV and RV types and their abbreviations. We believe their adoption will promote greater clarity and consistency in the terminology used in the scientific and medical literature. The recommendations will additionally provide a useful reference guide for journals, other publications and public databases seeking to use standardised terms for the growing multitude of enteroviruses and rhinoviruses described worldwide.

Enteroviruses and rhinoviruses are genetically related, but highly heterogeneous groups of viruses in both their disease associations in humans and in their antigenic characteristics. With such a vast collection of diverse viruses, virologists, infectious disease physicians and the wider medical community need names for enteroviruses that are clear, unambiguous and simple.

Genetic relationships of individual enteroviruses and rhinoviruses to each other and to other picornaviruses form the basis of their current taxonomy. Enteroviruses infecting humans are assigned to four species, *Enterovirus A – Enterovirus D*, and rhinoviruses are assigned to the species *Rhinovirus A – Rhinovirus C*, all in the genus *Enterovirus*. Numerous serologically distinct viruses (serotypes) have been assigned within each enterovirus and rhinovirus species.

Unfortunately, many of the naming conventions of enteroviruses are heavily influenced by a previously used and quite different biologically based classification system that cuts across their genetic relationships. Enteroviruses designated as coxsackieviruses were those able to infect newborn mice and were subdivided into groups A and B, depending on whether they caused flaccid or spastic paralysis. Coxsackie A viruses are, however, genetically highly heterogeneous and distributed across three different enterovirus species (*Enterovirus A, Enterovirus B* and *Enterovirus C*). The highly distinctive polioviruses, with their propensity to cause severe paralytic disease and permanent neurological damage, are now assigned to the species *Enterovirus C*, but this species includes several other serotypes with few if any known disease associations. The biological classification was discontinued in the late 1960s, and subsequently characterised new serotypes were named by consecutive numbering (enterovirus 68 –71). More recently, the numbers have been prefixed with A, B, C or D to indicate their species assignment.

Human rhinoviruses were originally assigned to approximately 100 consecutively numbered serotypes and assigned biologically to different groups based on their receptor use or antiviral sensitivity [1]. Assignment to major or minor receptor groups was subsequently shown to be partly associated with their genetic relationships. Rhinoviruses are now genetically assigned to the species *Rhinovirus A* (mostly major receptor group + minor receptor group) or *Rhinovirus B* (all major receptor group) on the basis of metrics of sequence divergence [6]. As with enteroviruses, a species letter prefix is now recommended to indicate these species assignments (*e.g.* RV-B2, RV-A16). Types assigned to a more recently established rhinovirus species, *Rhinovirus C*, are also numbered consecutively from RV-C1 to RV-C57.

Finally, the criteria that define individual enterovirus and rhinovirus types have changed, making their designations as “serotypes” obsolete. Originally, serotypes were defined by their antigenic and cross-neutralisation properties. However, it has since been demonstrated that different enterovirus serotypes consistently show greater than 25% nucleotide sequence divergence from each other in the VP1 gene, while variants of the same serotype show less than 25% divergence [6]. A lower nucleotide sequence divergence threshold of around 11% similarly divides human rhinoviruses into their previously designated serotypes [3]. These genetic thresholds are now used to define newly discovered types (rather than serotypes) in the absence of any antigenic characterisation.

Although assignments of enteroviruses and rhinoviruses to genera and species have been updated by the International Committee for Taxonomy of Viruses (ICTV) several times in recent years to better reflect their genetic relationships, the nomenclature of serotypes within species has changed very little. While this conservatism in nomenclature has advantages in maintaining consistency with the extensive literature on enteroviruses since the 1950s, a listing of viruses to their assigned species still appears haphazard and confusing (Table 1). There is, furthermore, an ongoing and pervasive inconsistency of enterovirus names in the literature. In the following list, we highlight a number of areas where poorly standardised or potentially illogical terminology and nomenclature have developed. We propose an internally consistent set of names and abbreviations to rectify this (recommendations underlined).

**Table 1:** Recommended names and abbreviations for enteroviruses and rhinoviruses^1^

Species	Enterovirus name	Abbreviation of enterovirus name
*Enterovirus A* (n = 25)	coxsackievirus A2 → A8, A10, A12, A14, A16	CVA2 → CVA8, CVA10, CVA12, CVA14, CVA16
	enterovirus A71, A76^2^, A89^2^ → A91, A114, A119^2^ → A121^3^	EV-A71, EV-A76, EV-A89 → EV-A91, EV-A114, EV-A119 → EV-A121
	Non-human enteroviruses^4^: enterovirus A92, A122 → A125 (formerly simian virus 19, 43, 46 and baboon enterovirus A13)	EV-A92, EV-A122 → EV-A125
*Enterovirus B* (n = 63)	coxsackievirus A9	CVA9
	coxsackievirus B1 → B6	CVB1 → CVB6
	echovirus 1 → 7, 9, 11 → 21, 24 → 27, 29 → 33	E1 → E7, E9, E11 → E21, E24 → E27, E29 → E33
	enterovirus B69, B73 → B75, B77 → B88, EV-B93, B97, B98, B100, B101, B106, B107^2^, B111^3^	EV-B69, EV-B73 → EV-B75, EV-B77 → EV-B88, EV-B93, EV-B97, EV-B98, EV-B100, EV-B101, EV-B106, EV-B107, EV-B111
	Non-human enteroviruses^4^: enterovirus B110, B112, B113, B114 (formerly simian virus SA5)	EV-B110, EV-B112 → EV-B114
*Enterovirus C* (n = 23)	coxsackievirus A1, A11, A13, A17, A19 → A22, A24	CVA1, CVA11, CVA13, CVA17, CVA19 → CVA22, CVA24
	enterovirus C95, C96, C99, C102, C104, C105, C109, C113, C116 → C118^3^	EV-C95, EV-C96, EV-C99, EV-C102, EV-C104, EV-C105, EV-C109, EV-C113, EV-C116 → EV-C118
	poliovirus 1 → 3	PV1 → PV3
*Enterovirus D* (n = 5)	enterovirus D68, D70, D94, D111^2, 3^	EV-D68, EV-D70, EV-D94, EV-D111
	Non-human enteroviruses enterovirus D120	EV-D120
*Rhinovirus A* (n = 80)	rhinovirus A1, A2, A7 → A13, A15, A16, A18 → A25, A28 → A34, A36, A38 → A41, A43, A45 → A47, A49 → A51, A53 → A68, A71, A73 → A78, A80 → A82, A85, A88 → A90, A94, A96, A100 → A109^5^	RV-A1, RV-A2, RV-A7 → RV-A13, RV-A15, RV-A16, RV-A18 → RV-A25, RV-A28 → RV-A34, RV-A36, RV-A38 → RV-A41, RV-A43, RV-A45 → RV-A47, RV-A49 → RV-A51, RV-A53 → RV-A68, RV-A71, RV-A73 → RV-A78, RV-A80 → RV-A82, RV-A85, RV-A88 → RV-A90, RV-A94, RV-A96, RV-A100 → RV-A109.
*Rhinovirus B* (n = 32)	rhinovirus B3 → B6, B14, B17, B26, B27, B35, B37, B42, B48, B52, B69, B70, B72, B79, B83, B84, B86, B91, B92, B93, B97, B99, B100 → B106^5^	RV-B3 → RV-B6, RV-B14, RV-B17, RV-B26, RV-B27, RV-B35, RV-B37, RV-B42, RV-B48, RV-B52, RV-B69, RV-B70, RV-B72, RV-B79, RV-B83, RV-B84, RV-B86, RV-B91, RV-B92, RV-B93, RV-B97, RV-B99, RV-B100 → RV-B106
*Rhinovirus C* (n = 57)	rhinovirus C1 → C57^6^	RV-C1 → RV-C57

^1^This list was compiled from http://www.picornaviridae.com/enterovirus/enterovirus.htm, which should be consulted for further information

^2^found also in non-human primates

^3^Enterovirus numbering up to 120 is interleaved, with types numbered sequentially irrespective of their species assignment (*e.g.*, EV-D68, EV-B69, EV-D70 and EV-A71 *etc.*). Beyond 120, numbering is non-interleaved, with EV-A121 and future assignments of EV-B121, EV-C121 *etc.* representing different types

^4^Previous names of non-human primate enteroviruses are in parentheses

^5^Rhinovirus numbering for members of the species *Rhinovirus A* and *Rhinovirus B* is interleaved (see footnote 3), but non-interleaved after 100, so that RV-A101 and RV-B101 are separate types

^6^Numbering for members of the species *Rhinovirus C* has always been non-interleaved, with current type assignments 1 to 57 representing different types from those numbered 1-57 in the species *Rhinovirus A* and *Rhinovirus B*

- Enteroviruses infecting humans are assigned to the species *Enterovirus A – Enterovirus D*, and rhinoviruses to *Rhinovirus A – Rhinovirus C.*The names of species and of other taxa are italicised and capitalised and cannot be abbreviated.
- Names for groups of viruses, such as “picornaviruses” or “enteroviruses” or even “species A enteroviruses” are acceptable for descriptive purposes. However, these names are not substitutes for the names of taxa. For example, the term “enteroviruses” refers to a group of actual viruses, while the corresponding taxon, *Enterovirus*, is the genus to which enteroviruses are assigned.
- Names of viruses, as opposed to species, should not be capitalised, so the terms enterovirus, rhinovirus, coxsackievirus, poliovirus *etc.* are correct. Note, however, that the name of coxsackieviruses derives from the name of a town, Coxsackie, in New York State, where it was first isolated. While coxsackievirus names were originally capitalised for this reason, this is no longer recommended.
- Enteroviruses characterised after 1967 were named numerically, originally as EV68, EV69, EV70 and EV71 [4, 5]. All newly identified enteroviruses since then have also been numbered sequentially (Table 1). We endorse the previous recommendation from the*Picornaviridae*Study Group that these names should additionally include a letter corresponding to their species assignment along with hyphenation. These four enterovirus examples now bear the names EV-D68, EV-B69, EV-D70 and EV-A71.
- The retention of “coxsackievirus” in the name of various enteroviruses has recently produced considerable nomenclatural inconsistency and drawing of false analogies. Abbreviation formats in the current literature for the medically important coxsackievirus A16 (a frequent aetiological agent of hand, foot and mouth disease [HFMD]) belonging to the species *Enterovirus A* include “CAV16”, “CAV-16”, “CVA16”, “CVA-16” and “CV-A16”. The latter format is increasingly used in the literature; we believe this is largely motivated by analogy to the name format of another major cause of HFMD, EV-A71 (see section d). By extension, names for other coxsackie A virus are frequently abbreviated in the same way (CV-A6, CV-A10, *etc.*).
  The problem with this practice is that, while seemingly helpful as a means to indicate the species in the above examples, there are several coxsackie A viruses in the species *Enterovirus C* (CVA1, CVA11, CVA13, CVA17, CVA19, CVA20, CVA21, CVA22, CVA24) and one in the species *Enterovirus B* (CVA9; Table 1). To abbreviate these as “CV-A1”, “CV-A11”, and “CV-A9” would imply to many who are used to the EV-A71 (and CV-A16) nomenclature that these viruses also belong to species A.
  We recommend that the abbreviations of coxsackieviruses are not hyphenated to emphasise the difference from enterovirus names,*e.g.*CVA16, CVA9, CVA21.
- Echoviruses were named after the abbreviation enteric cytopathic human orphan viruses, with the “orphan” part reflecting a belief at a time in the late 1950s that their infections were not associated with disease. While the older original literature referred to them as ECHO virus 6 or ECHO virus 11, the abbreviation “ECHO” is typically no longer capitalised (echovirus 6, echovirus 11). How these names are further abbreviated is highly variable in the literature, with the range of terms for echovirus 6 (as an example) including “echo6”, “Echo6”,” Echo 6”, “EV6”, “E-6” and “E6” co-existing in the current literature. For clarity, we recommend the last form (E6).
- The names of rhinovirus types should be numbered sequentially with a hyphen and species prefix to the type number, comparable to the format of enterovirus names (Table 1).
- The prefix “human” should not be used in the names of enterovirus or rhinovirus taxa or in their individual names or abbreviations. Particularly misleading is the abbreviation “HEV” for human enterovirus, given its wider and standard use for hepatitis E virus.
- Several enteroviruses have been detected and characterised in non-human primates (*e.g.*, EV-A92, simian virus 5, baboon enterovirus A13 and simian enterovirus SA5), while others have been isolated from humans and apes (*e.g.,* EV-A119, EV-D111) (Table 1). Their current nomenclature is internally inconsistent, some being assigned in the same number series used for human enteroviruses and others possessing names reflecting their (non-human) host. We recommend that these be renamed according to the serial type numbering convention used for enteroviruses as described in Section d (proposed names and abbreviations are listed in Table 1).
- Newly characterised enteroviruses and rhinoviruses have been classified based on genetic relationships, and information on their antigenic (cross-neutralisation) characteristics is not often available. We recommend that the term “type” rather than “serotype” be used for the nomenclature of all enteroviruses and rhinoviruses.

The ICTV remit of the Study Group extends down only to the rank of species assignments and nomenclature. However, as an expert group, we have extensively advised on nomenclatural conventions for enteroviruses and rhinoviruses (and other picornaviruses) and the assignment of a uniform numbering for types within each. The development of community-adopted guidelines, based around the issues described above, have been of considerable value for naming standardisation and their representation in public databases and the literature. Although not part of these recommendations, we note a change in the nomenclature and abbreviations for human parechoviruses (members of the species *Parechovirus A*). There is increasing use of the abbreviations PeV-A1 and PeV-A3 for parechovirus types 1 and 3 (formerly abbreviated as HPeV-1 and HPeV3) which indeed parallels the changes in nomenclature of EV and RV types recommended above.

More-radical revision of enterovirus names was discussed previously as genetic relationships of enteroviruses became apparent, including the proposal that all enteroviruses be numbered sequentially and that the biologically derived names such as “coxsackievirus”, “echovirus” and “poliovirus” be discontinued [2]. However, even in its publication year of 1962, it was generally believed that the biological classification of enteroviruses was so entrenched that these terms should not be abandoned. Nearly 60 years later, we similarly accept the need for historical continuity of virus names despite its attendant intrinsic complexities. We hope, however, that this proposal for an agreed upon and consistent set of names and abbreviations of enteroviruses and rhinoviruses will bring some clarity for medical and scientific practitioners in this area.

Both the *Picornaviridae* Study Group and the ICTV maintain an up-to-date list of currently assigned enterovirus and rhinovirus types:

http://www.picornaviridae.com/enterovirus/enterovirus.htm

https://ictv.global/report/picornaviridae/enterovirus/

These reflect the nomenclature recommendations in the current communication and contain further information on other enteroviruses and wider classification. The recommendations are endorsed by the European Non-Polio Enterovirus Network (ENPEN) and we acknowledge the helpful review from their members.
