# Correction to: Recommendations for the nomenclature of enteroviruses and rhinoviruses

**DOI:** 10.1007/s00705-020-04558-x

**Published:** 2020-03-23

**Authors:** P. Simmonds, A. E. Gorbalenya, H. Harvala, T. Hovi, N. J. Knowles, A. M. Lindberg, M. S. Oberste, A. C. Palmenberg, G. Reuter, T. Skern, C. Tapparel, K. C. Wolthers, P. C. Y. Woo, R. Zell

**Affiliations:** 1 Nuffield Department of Experimental Medicine, University of Oxford, Oxford, UK; 2 Department of Medical Microbiology, Leiden University Medical Center, Leiden, The Netherlands; 3 National Microbiology Service, NHS Blood and Transplant, London, UK; 4 Finnish Institute for Health and Welfare, Helsinki, Finland; 5 The Pirbright Institute, Pirbright, Woking, UK; 6 Department of Chemistry and Biomedical Sciences, Linnaeus University, Kalmar, Sweden; 7 Division of Viral Diseases, Centers for Disease Control and Prevention, Atlanta, GA USA; 8 Department of Biochemistry, Institute for Molecular Virology, Madison, WI USA; 9 Department of Medical Microbiology and Immunology, Medical School, University of Pécs, Pecs, Hungary; 10 Max Perutz Labs, Medical University of Vienna, Vienna, Austria; 11 Department of Microbiology and Molecular Medicine, Faculty of Medicine, University of Geneva, Geneva, Switzerland; 12 Department of Medical Microbiology, Academic Medical Center, University of Amsterdam, Amsterdam, The Netherlands; 13 Department of Microbiology, The University of Hong Kong, Hong Kong SAR, China; 14 Division of Experimental Virology, Institute of Medical Microbiology, Jena University Hospital, Jena, Germany; 15 Faculty of Bioengineering and Bioinformatics, Lomonosov Moscow State University, Moscow, Russia; 16 Department of Biomedical Data Sciences, Leiden University Medical Center, Leiden, The Netherlands

## Correction to: Archives of Virology 10.1007/s00705-019-04520-6

Unfortunately, two of the affiliations of author “A. E. Gorbalenya” were missed in original version. The affiliation is updated here.

“*Faculty of Bioengineering and Bioinformatics, Lomonosov Moscow State University, Moscow, Russia*”

“*Department of Biomedical Data Sciences, Leiden University Medical Center, Leiden, The Netherlands*”

